# Reversible azoospermia associated with the selective JAK1 inhibitor abrocitinib: a case report

**DOI:** 10.1007/s10815-026-03993-0

**Published:** 2026-08-05

**Authors:** Giuseppe Seminara, Rossella Cannarella, Aldo Eugenio Calogero, Antonio Aversa

**Affiliations:** 1 Endocrinology Unit, Renato Dulbecco University Hospital, Catanzaro, Italy; 2 Department of Experimental and Clinical Medicine, Magna Graecia University, Catanzaro, Italy; 3 Department of Clinical and Experimental Medicine, University of Catania, Catania, Italy

**Keywords:** Atopic dermatitis, JAK inhibitors, Azoospermia, Male infertility

## Abstract

**Objective:**

To describe a case of reversible azoospermia temporally associated with abrocitinib treatment in a man with severe atopic dermatitis (AD) and discuss its potential implications for male reproductive safety.

**Design:**

Case report.

**Patient:**

A 38-year-old normal-weight man with highly refractory AD, a history of neonatal orchidopexy, and primary infertility.

**Intervention:**

Clinical, hormonal, and semen evaluation before and after discontinuation of abrocitinib therapy (200mg/day).

**Main outcome measures:**

Semen parameters and reproductive hormone profile.

**Results:**

Two consecutive semen analyses performed during abrocitinib treatment demonstrated azoospermia. Baseline endocrine evaluation showed elevated follicle-stimulating hormone (FSH; 12.3 mIU/mL), suggestive of seminiferous tubule dysfunction. Abrocitinib was discontinued, and reassessment after 3 months revealed complete recovery of spermatogenesis, with a sperm concentration of 50 million/mL. Although isolated teratozoospermia persisted, FSH levels normalized (8.6 mIU/mL) and inhibin B increased markedly (from 58 to 135 pg/mL), consistent with improved Sertoli cell function. The temporal association between drug withdrawal and recovery supports a potential reversible effect on spermatogenesis.

**Conclusion:**

This is, to our knowledge, the first report describing reversible azoospermia temporally associated with abrocitinib therapy. Although causality cannot be established from a single observation, this case raises a potential safety signal regarding the effects of selective JAK1 inhibition on male reproductive function. Further preclinical studies, pharmacovigilance analyses, and prospective clinical investigations are warranted to determine whether prolonged or high-dose abrocitinib exposure may adversely affect spermatogenesis in susceptible individuals.

## Introduction

The landscape of management for moderate-to-severe atopic dermatitis (AD) has been significantly reshaped by the introduction of targeted small-molecule inhibitors, specifically Janus kinase (JAK) inhibitors [1]. Among these, abrocitinib stands out as an oral, once-daily, highly selective JAK1 inhibitor that downregulates the signaling of pro-inflammatory cytokines involved in AD pathogenesis, including interleukin (IL)−4, IL-5, IL-13, and IL-31 [2]. While its efficacy and systemic safety profile have been extensively validated in multi-center clinical trials [3, 4], our understanding of its potential off-target effects on the male reproductive system remains limited.

Spermatogenesis is a highly coordinated and complex biological process that relies heavily on precise endocrine and paracrine signaling networks. Accumulating evidence indicates that the Janus kinase/signal transducer and activator of transcription (JAK-STAT) pathway plays a pivotal role in maintaining testicular homeostasis [5, 6]. Within the testicular niche, cytokines and growth factors—most notably glial cell line-derived neurotrophic factor (GDNF)—signal through the JAK-STAT pathway, specifically activating STAT3, to regulate the self-renewal, proliferation, and differentiation of spermatogonial stem cells (SSCs) [7, 8]. Furthermore, JAK-STAT signaling is intricately involved in gonocyte-Sertoli cell survival [9], as well as in the follicle-stimulating hormone (FSH) signaling pathway, as recently documented in granulosa cells [10]. Consequently, pharmacological disruption of this pathway via selective JAK inhibition poses a theoretical risk of impairing the stem cell niche or disrupting the later stages of germ cell maturation.

While preclinical reproductive toxicity studies for some JAK inhibitors have shown transient changes in semen parameters or fertility indices at supratherapeutic doses [11], robust clinical data regarding the impact of selective JAK1 inhibitors on human spermatogenesis are remarkably scarce. To date, abrocitinib-associated drug-induced azoospermia remains poorly characterized in the clinical literature.

Herein, we report a compelling case of a patient with AD who developed azoospermia during abrocitinib therapy, which fully reversed after its discontinuation.

## Case report

A 38-year-old male patient presented to our Andrology Outpatient Clinic in February 2026, complaining of a 1-year history of secondary infertility with his current 36-year-old female partner, who had no significant medical conditions. His presentation followed a sequential diagnostic timeline initiated earlier that year to investigate the couple’s fertility status. Notably, the patient had a proven history of fertility, having previously fathered two children with a former partner over one decade ago. His personal medical history was significant for a right orchidopexy performed in the neonatal period for cryptorchidism. He reported a chronic smoking habit of approximately 20 cigarettes per day, but denied any history of alcohol abuse or recreational drug use. Family history was notable for type 2 diabetes mellitus and Alzheimer’s disease. The patient suffered from severe atopic dermatitis that was highly refractory to conventional topical and systemic therapies, prompting the oral selective JAK1 inhibitor abrocitinib at a dose of 100 mg daily in February 2025. Due to an insufficient clinical response, the dosage was escalated to 200 mg daily 4 months later, in June 2025. Immediately, prior to his andrological evaluation in February 2026, the patient abruptly discontinued abrocitinib therapy due to an acute onset of herpetic keratitis, which was being actively treated with systemic and topical antiviral therapy. From a general clinical standpoint, the patient was in good health and had no other chronic conditions other than atopic dermatitis and an acute herpetic infection. Anthropometric assessment indicated a normal-weight individual, with a body mass index (BMI) of 24.3 kg/m^2^.

The clinical investigation followed a precise timeline: it was initiated after the documentation of azoospermia in an initial screening semen analysis performed in mid-January 2026. To confirm this critical finding, a second consecutive semen analysis was executed in early February 2026, approximately 3 weeks later and just prior to his formal andrological consultation. To ensure maximum diagnostic accuracy, reproducibility, and compliance with international standards, all seminal fluid evaluations across the timeline of this case were conducted at our institutional laboratory in strict accordance with the World Health Organization laboratory manual for the examination and processing of human semen [12]. Each specimen was obtained by masturbation directly into a sterile plastic container after an explicitly enforced sexual abstinence period of 3 to 5 days. Following collection, the samples were immediately incubated at 37 °C for 45 min to allow complete liquefaction. Standardized macroscopic examination was then performed to evaluate ejaculate volume, pH, appearance, and viscosity. At baseline (prior to abrocitinib discontinuation), all macroscopic parameters were within normal reference limits (including a baseline semen volume of 2.5 mL, normal pH, normal longitudinal viscosity, and a typical opalescent/milky-white appearance). Notably, semen volume and color remained unchanged before and after abrocitinib discontinuation, showing complete stability across the entire timeline of the clinical investigation. For the microscopic assessment, initial wet preparations of 10 µL of well-mixed semen were examined under phase-contrast microscopy at × 400 magnification to evaluate the presence of spermatozoa. Given the apparent total absence of germ cells on the initial wet mounts, a rigorous centrifugation protocol was mandatory to confirm true azoospermia and rule out cryptozoospermia. Both baseline analyses consistently demonstrated the absolute absence of spermatozoa in the entire pellet, confirming a diagnosis of azoospermia.

Concurrently, a physical and instrumental diagnostic workup was performed. Scrotal ultrasonography revealed significant testicular asymmetry, with a left testicular volume of 14 mL and a hypotrophic right testis measuring 6 mL. The right testis displayed a mildly inhomogeneous echotexture but was entirely free of focal lesions, cysts, or neoplastic masses, and no clinical or subclinical varicocele was detected bilaterally.

Blood samples were strictly obtained in the morning, between 08:00 AM and 09:30 AM, following a complete overnight fast of at least 8 h. The samples were immediately dispatched to the centralized laboratory of the “Renato Dulbecco” University Hospital of Catanzaro. Serum concentrations of total testosterone, FSH, luteinizing hormone (LH), prolactin, and thyroid-stimulating hormone (TSH) were quantified via ADVIA Centaur XP® (Siemens Healthcare Diagnostics Inc., Tarrytown, NY, USA), whereas serum inhibin B was measured using a commercially available enzyme-linked immunosorbent assay (ELISA) kit. The baseline endocrinological profile indicated a euthyroid state and normal prolactin levels, accompanied by a total testosterone level of 413 ng/dL, a LH level of 9.0 mIU/mL, and an elevated FSH level of 12.3 mIU/mL, which reflected a state of seminiferous tubule dysfunction. Notably, the baseline inhibin B level was reduced at 58 pg/mL (normal reference range, 100–400 pg/mL), providing additional biochemical evidence of Sertoli cell dysfunction and germinal epithelium impairment.

The patient was subsequently scheduled for a follow-up assessment in May 2026, marking exactly three months of complete abrocitinib discontinuation. A repeated hormonal assessment demonstrated a notable biochemical recovery, characterized by a reduction of the FSH level to a borderline value of 8.6 mIU/mL, an LH level of 5.9 mIU/mL, an increase in total testosterone to 646 ng/dL, and a recovery of inhibin B levels, which rose to 135 pg/mL.

Crucially, a follow-up semen analysis was executed using the same standardized laboratory methodology and strict abstinence criteria. The microscopic evaluation revealed a restoration of spermatogenesis, with a semen volume of 2.6 mL, a sperm concentration of 50 million/mL, and a total sperm count of 130 million. The kinetic evaluation revealed a progressive motility of 42% and a non-progressive motility of 16%, yielding a total motility of 58%, while 42% of the observed spermatozoa were immotile. Sperm morphology revealed that only 3% of the cells exhibited normal head, neck, and tail structures. Consequently, the patient’s seminal profile demonstrated a transition from absolute azoospermia to a phenotype of isolated teratozoospermia.

**Fig. 1:**
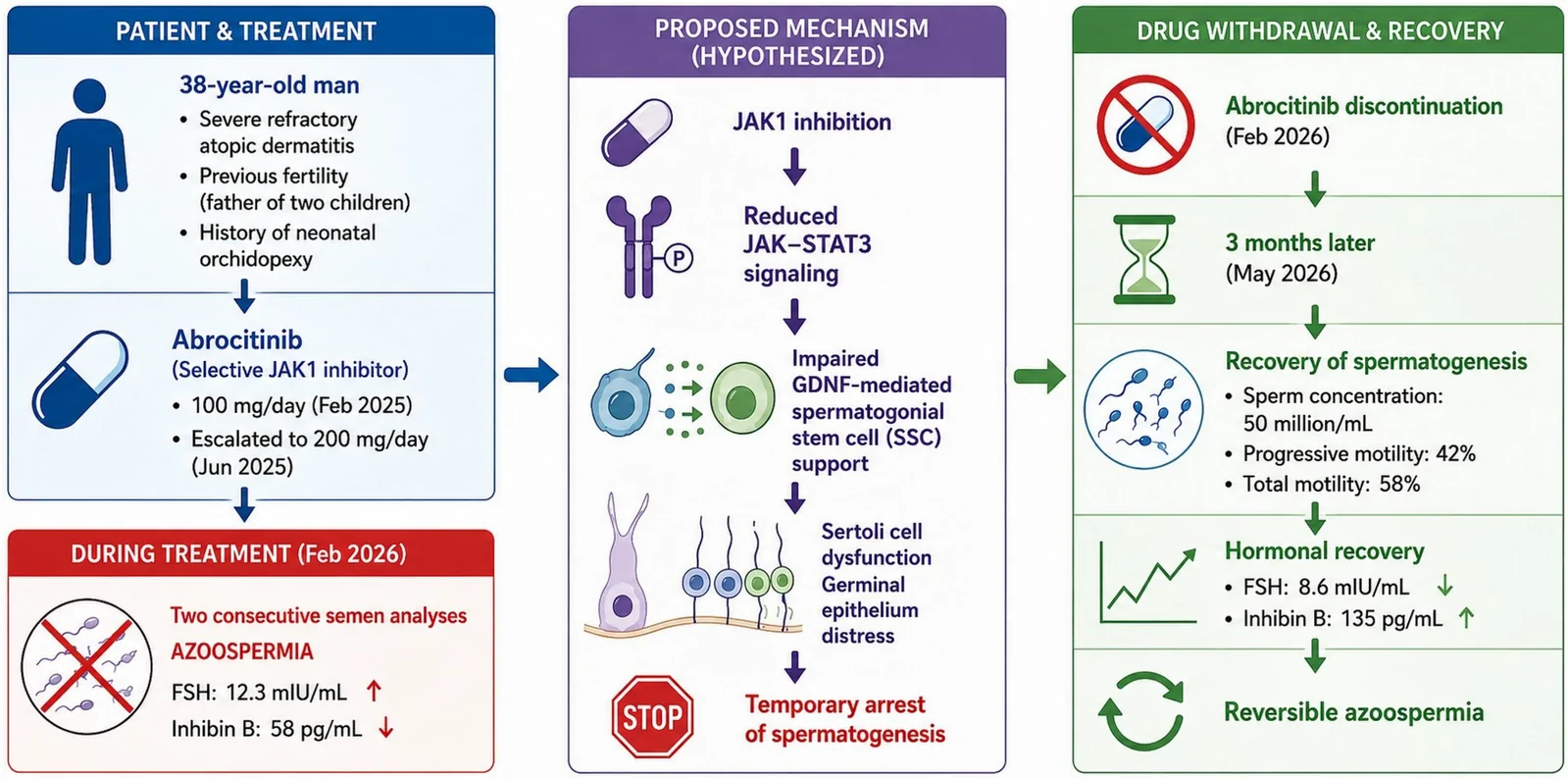
Temporal association between abrocitinib therapy and reversible azoospermia. High-dose selective JAK1 inhibition was associated with spermatogenic arrest, reduced inhibin B levels, and elevated FSH, followed by recovery of sperm production and testicular function after treatment withdrawal

A graphical summary of the present case is included in Fig. 1.

## Discussion

To the best of our knowledge, the present case represents the first report in the medical literature describing reversible azoospermia temporally associated with treatment with the selective JAK1 inhibitor abrocitinib. Indeed, the potential impact of targeted therapies on male fertility is becoming an increasingly recognized and debated issue in contemporary clinical practice [13]. While drug-induced reversible azoospermia is an extremely rare phenomenon that has been documented for only a few pharmacological agents [14–16], it has never been previously described in humans in association with selective JAK1 inhibitors. Although the clinical efficacy and systemic safety profile of this small molecule have been extensively validated and documented in large phase 3 randomized controlled trials [3, 4], these studies did not include semen analysis or the evaluation of male fertility parameters among their safety outcomes. Consequently, the potential impact of abrocitinib on the human germline has remained uninvestigated, resulting in a knowledge gap in the long-term management of male patients of childbearing age.

This lack of clinical data on male reproductive function contrasts, at least in part, with the preclinical and clinical evidence available for other JAK inhibitors, revealing significant differences in the scope and methodology of their reproductive toxicity evaluations. For instance, the preclinical scope for filgotinib, another selective JAK1 inhibitor, explicitly included robust male animal toxicology models (specifically in rats and dogs), which methodologically evaluated direct testicular parameters, demonstrating a clear dose-dependent adverse effect characterized by testicular atrophy, reduced cellularity of the seminiferous tubules, and germ cell degeneration [11]. These preclinical safety signals prompted regulatory authorities to mandate dedicated, prospective, randomized phase 2 clinical trials in humans, known as the MANTA and MANTA-Ray studies [17]. Although these human trials, conducted in patients with chronic inflammatory bowel and rheumatic diseases, ultimately showed no statistically or clinically significant impact of filgotinib on spermatogenesis or testicular endocrine function, they represent a highly structured, prospective approach to human male reproductive safety [17]. In stark contrast, the scope of preclinical reproductive toxicological studies for abrocitinib was primarily restricted to evaluating female animal models, demonstrating an ability to induce reversible infertility and embryo-fetal lethality [18]. Furthermore, the clinical development methodology for abrocitinib did not include any prospective semen analyses or dedicated male fertility evaluations within its large phase 3 randomized controlled trials [3]. Consequently, the absence of comparable prospective studies evaluating male reproductive function underscores a critical gap in its safety evaluation, making the findings of our case report particularly relevant from a pharmacovigilance perspective.

From a pathophysiological standpoint, several molecular mechanisms underlying the observed spermatogenic arrest can be hypothesized. The striking temporal coincidence between the detection of azoospermia during therapy with abrocitinib, followed by the reappearance of spermatozoa in the ejaculate exactly three months after drug discontinuation, raises the hypothesis of a potential, reversible gonadotoxic effect on the germline, although a definitive causal relationship cannot be definitively proven. Biochemically, abrocitinib selectively blocks the JAK1 pathway, disrupting the subsequent phosphorylation and activation of the STAT3 transcription factor [19]. Within the testicular microenvironment, the JAK-STAT3 signaling pathway is fundamental for mediating the paracrine effects of cytokines and growth factors, most notably GDNF [7, 8]. The GDNF/JAK/STAT3 axis is an indispensable regulator of SSC niche homeostasis, governing the delicate processes of self-renewal, proliferation, and differentiation of SSCs [7, 8]. Chronic pharmacological inhibition of this pathway could therefore lead to a transient maturation arrest of spermatogonia or alter the biochemical support provided by Sertoli cells, thereby compromising the integrity of the blood-testis barrier and gonocyte survival [9, 20]. This pathophysiological cascade is further corroborated by the longitudinal fluctuations of inhibin B observed in our patient. The baseline reduction of inhibin B serves as a reliable proxy for abrocitinib-induced spermatogenic arrest and Sertoli cell impairment. Conversely, its subsequent rebound into the normal range closely tracks the restoration of sperm concentration, highlighting the reversible nature of this selective JAK1-mediated gonadotoxic effect. Notably, recent evidence has shown that the JAK pathway is involved in FSH-mediated signaling in granulosa cells [10]. Given the conserved molecular mechanisms shared between granulosa and Sertoli cells [21], a potential role of JAK signaling in mediating the molecular effects of FSH in Sertoli cells cannot be excluded, further explaining the findings herein observed and the increase in FSH levels.

In addition to the intrinsic biological action of the drug, the patient’s peculiar individual susceptibility must also be considered. Specifically, the history of a right orchidopexy performed during the neonatal period for cryptorchidism likely accounts for why his serum FSH levels returned to a borderline-high value (8.6 mIU/mL) rather than a fully optimal baseline after abrocitinib cessation. Unilateral cryptorchidism, even when corrected early in life, is frequently associated with long-term microstructural anomalies, subclinical germinal epithelium distress, and a smaller total Sertoli cell pool that can also subclinically affect the contralateral descended testis. In this patient, such underlying impairment is clinically evidenced by the severe right testicular hypotrophy (6 mL). Therefore, as previously suggested, this history points to a pre-existing vulnerability and a chronically reduced functional resilience of the seminiferous epithelium. While the abrupt post-withdrawal drop in FSH directly reflects the elimination of the drug-induced gonadotoxic insult, the persistent borderline elevation represents the patient’s intrinsic andrological baseline, which likely also heightened his parenchyma’s susceptibility to high-dose pharmacological exposure in the first place.

When interpreting these findings, the limitations inherent to a single case report must be acknowledged, as the temporal association observed does not establish causality and may be influenced by potential confounding factors.. The patient was a heavy chronic smoker of approximately 20 cigarettes per day, a habit notoriously correlated with a worsening of semen quality; however, his prior and documented natural fertility with a former partner suggests that tobacco use cannot be considered the primary etiological factor for the sudden and profound spermatogenic arrest. Similarly, the acute onset of herpetic keratitis occurred only in conjunction with our final evaluation in February 2026; although severe systemic viral infections and acute febrile states can transiently depress spermatogenesis, the strictly localized and non-systemic nature of the ocular pathology makes its causal role in the azoospermia—which had already been established in the preceding months—extremely unlikely.

In conclusion, while this single clinical observation cannot replace epidemiological data derived from large-scale prospective cohort studies, the temporal alignment between drug discontinuation and the recovery of spermatogenesis—which closely mirrors the chronological duration of the human spermatogenic cycle—represents a noteworthy observation. Rather than establishing a definitive class effect, this evidence serves as a preliminary signal that merely suggests the opportunity to consider reproductive safety in male patients of childbearing age who are candidates for prolonged or high-dose therapies with selective JAK1 inhibitors. Further prospective surveillance and registry data are strictly required to clarify whether this phenomenon represents an under-recognized drug-induced effect or an isolated, idiosyncratic response, before systematic recommendations or routine semen analysis monitoring can be formally integrated into clinical guidelines.

## Data Availability

No datasets were generated or analysed during the current study.
